# Impedance Spectroscopy Analysis and Equivalent Circuit Modeling of Graphene Oxide Solutions

**DOI:** 10.3390/nano7120446

**Published:** 2017-12-14

**Authors:** Youngbin Yoon, Jeonghoo Jo, Seungdu Kim, In Gyu Lee, Byung Jin Cho, Myunghun Shin, Wan Sik Hwang

**Affiliations:** 1 Department of Materials Engineering, Korea Aerospace University, Goyang 10540, Korea; ybyoon93@gmail.com (Y.Y.); seungdukim@gmail.com (S.K.); leeig@kau.ac.kr (I.G.L.); 2 School of Electronics and Information Engineering, Korea Aerospace University, Goyang 10540, Korea; jhjo@kau.kr; 3 Department of Electrical Engineering, KAIST, Daejeon 34141, Korea; elebjcho81@kaist.ac.kr

**Keywords:** graphene oxide solution, impedance spectroscopy analysis, equivalent circuit model

## Abstract

The optical and electrical characteristics of a graphene oxide solution (GS) with different graphene oxide (GO) concentrations in de-ionized water are investigated via the electrochemical impedance spectroscopy (EIS) method. The measurement results produced by the EIS for the GS are represented with both Bode and Nyquist plots in a frequency range from 1 kHz to 10 MHz. Using these results, we develop an equivalent circuit model as a function of the GO concentration, representing the GS as a mixed circuit of two-dimensional (2D) GO dispersed in parallel in de-ionized (DI) water. The underlying physics of the current-flowing behavior in the GS are explained and interpreted using empirical circuit models; the circuit model also shows that highly resistive GO becomes conductive in GS form in the DI water. The findings in this work should draw new attention toward GSes and related applications, including functional composite materials, catalysts, and filter membranes.

## 1. Introduction

Graphene has been intensively investigated for various applications due to its excellent electrical, mechanical, thermal, and optical properties [1,2,3,4], since its re-discovery using Scotch Tape in 2004. Though this early-stage graphene was initially produced via the mechanical exfoliation approach (Scotch Tape method), at present, most graphene is prepared via the chemical vapor deposition (CVD) method, which provides substantial benefits in terms of the large-scale processing of wafer size. However, CVD graphene requires a very high temperature and transferring processes when it is deposited onto the desired substrate, which is not suitable for commercialized graphene-embedded products. Alternatively, graphene oxide (GO) dispersed in a solvent could be a promising method for wafer-size mass production at low costs [5,6]. GO can be easily coated on a target substrate using spin-coating and/or spray methods as a pre-cursor, and then turned into graphene via thermal, chemical, or photocatalytic reduction [7]. Meanwhile, GO is not only used as a precursor for grapheme, but also performs its own functions with a hydroxyl/epoxide group on the basal plane and carbonyl/carboxyl group on the edge, which is unlike the finally-produced grapheme [8,9]. Due to such oxygen-containing functional groups, GO shows excellent chemical sensing properties [10] and can be dispersed uniformly and stably in the de-ionized (DI) water (hereinafter referred to as a graphene oxide solution (GS)) [11]. At present, GSes are commercially available for various applications, such as transparent conductive coatings, polymer synthesis, ultra-strong graphene oxide paper, supercapacitors, solar cells, electro-static dissipation (ESD) films, chemical and bio sensors, and the purification of wastewater [12,13,14,15,16,17,18,19,20]. Despite these numerous advantages, the detailed electrical characteristics of GSes have not been fully reported yet for different GO concentrations.

This study investigated GS electrical characteristics for different GO concentrations. In detail, electrochemical impedance spectroscopy (EIS) was performed, which was known to be a suitable technique to investigate the electrical properties of liquid materials. The observed experimental results were correlated with the equivalent circuit modeling at different GSes, which enabled the physical parameters to be extracted to explain and interpret the current-flowing mechanism of the various GSes. The findings in this work should draw new attention toward GSes and their related applications, including functional composite materials, catalysts, and filter membranes.

## 2. Experiment

The GO was obtained from graphite (−200 mesh, 99.9999%, Alfa Aesar, Ward Hill, MA, USA) via the Hummers method [21,22]. The residual metal ions and acids in the GO were removed using a filter press system, which was well described in the previous report [23]. After the purification process, the residual impurities in the GO were measured via an X-ray photoelectron spectroscopy (XPS) analysis. The results showed that K and Mn were not detected on the GO, but 1.1 percent of atomic S was observed. Based on this XPS analysis, a very small amount of S remained on the GO, and the S was not removed, even after the washing process was carried out five times. This indicated that the residual S was strongly attached to the GO flakes, and the impedance effect would be negligible. Next, the purified GO was freeze-dried, turned into powder form, and subsequently dispersed in DI water via sonication. Finally, a stable, homogeneous, dark-brown GS formed. This type of GS is known to be stable, even after almost two years of aging [24]. For the experiment, various GS samples with different GO concentrations were produced by mixing the predetermined 10 g/L GS (10 g GO per 1 L DI water) with DI water at different ratios.

**Figure 1:**
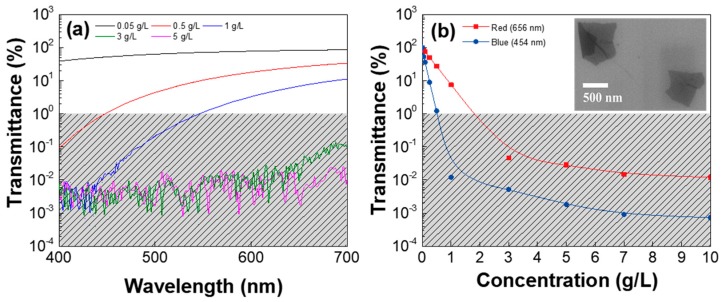
Transmittance of different GSes (**a**) as a function of wavelength and (**b**) as a function of graphene oxide (GO) concentration in the graphene oxide solution (GS) at 454- and 656-nm wavelengths. The inset shows a typical scanning electron microscope (SEM) image of the GO flakes on the SiO_2_/Si wafer.

Figure 1a shows the transmittance of the different GSes (different GO concentrations) as a function of wavelength in a visible wavelength range from 400 to 700 nm. The GS with a low GO concentration of 0.05 g/L was somehow transparent in the visible wavelength, but the GS became obviously opaque as the GO concentration increased over 0.5 g/L. Collectively, the transmittance of the GS at the 454- and 656-nm wavelengths is shown as a function of GO concentration in Figure 1b. The transmittance of the GS decreased exponentially as the GO concentration increased, and the GS became completely opaque when the GO concentration exceeded 0.5 g/L at 454 nm (2 g/L at 656 nm). The transmittance of the GS with the 10-g/L GO concentration was 10^5^ times smaller than that of the DI water.

For convenience in the discussion below, a sample number of GSes was assigned according to GO concentrations, as shown in Table 1; GS1 and GS20 were dispersed in the 0.25 g GO and 7.00 g GO, respectively, in 1 L of DI water. In addition, the GS samples were classified into two groups—a relatively low GO concentration (GS1 to GS11) and relatively high GO concentration (GS12 to GS20), which is subjective. The electrical impedances of the different GSes (from the DI water and GS1 to GS20) were measured using 24 × 24-mm^2^ parallel copper electrodes 20 mm apart. The copper plates were used as electrodes due to the ease of integration into the homemade measurement system for this study. The measuring frequency range was 1 kHz to 10 MHz, with an AC voltage of 30 mV using a Keithley 4200-SCS parameter analyzer, indicating that interface information between the electrode and solution was excluded.

## 3. Results and Discussion

The measured electrical impedance (*Z*) was rendered in a complex Cartesian form (*Z* = *Z*_Re_ + *jZ*_Im_), where *Z*_Re_ and *Z*_Im_ were the real (resistance) and imaginary (reactance) portions of the impedance. The real portion in the low frequency range strongly depended on the resistor value measured in DC mode while the imaginary portion was related to the reactance of the equivalent circuit formed between the parallel copper electrodes, and it may have varied greatly with respect to the frequency depending on the equivalent circuit structure.

**Figure 2:**
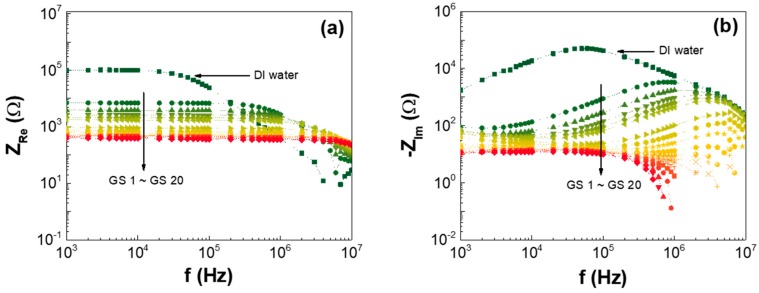
Frequency response of the (**a**) real and (**b**) imaginary portions of the GS impedances at different GSes.

Figure 2a,b presents the frequency responses of the real and imaginary portions of the impedances of the different GSes listed in Table 1. These results showed that both the real and imaginary components of the impedances decreased as the GO concentration increased in the GS. As shown in Figure 2a, the resistance of the GS decreased as the GO increased.

**Figure 3:**
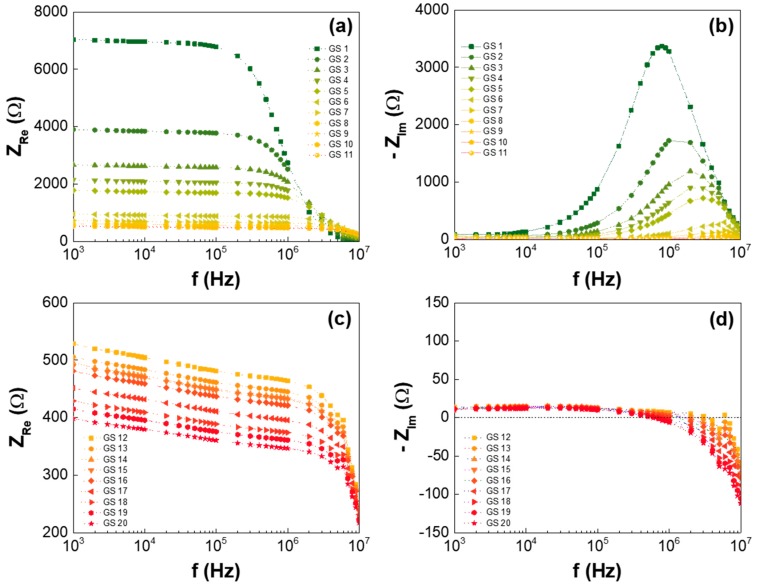
Frequency response of GS1 to GS11: (**a**) real and (**b**) imaginary portions of the impedance, and frequency response of GS12 to GS20: (**c**) real and (**d**) imaginary portions of the impedance.

In detail, the frequency responses of the real and imaginary portions of the GS impedance are shown with a linear scale in Figure 3. (Figure 3a,b is for GS1 to GS11, and Figure 3c,d is for GS11 to GS20.) As discussed in Figure 2, the resistance of the various GSes (GS1 to GS20) decreased as the GO concentration in the GS increased, and the frequency improved for the GSes with both the low and high GO concentrations. This implies that both the resistive and capacitive paths for the current-flow existed in the GS. In Figure 3b, the reactance of the GSes with low GO concentrations showed the rollover for the frequency, representing a capacitive-mode behavior. However, the reactance of the GSes with high GO concentrations steadily declined at high frequencies, indicating that the capacitive-mode behavior shifted to inductive-mode behavior, as shown in Figure 3d. This reveals that the current-flowing mechanism in the GS involved inductor components for GSes with high GO concentrations and high frequency regions.

**Figure 4:**
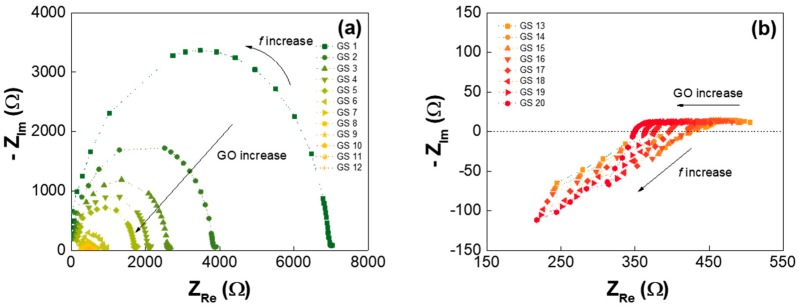
Nyquist plot of the GS samples: (**a**) for GS1 to GS11, which have low GO concentrations, and (**b**) for GS12 to GS20, which have high GO concentrations.

This impedance changes with frequencies can be also interpreted with a Nyquist plot where the impedance is plotted with Cartesian coordinates of the real portion on the *X*-axis and the imaginary portion on the *Y*-axis. Figure 4 shows the Nyquist impedance plots of the two GS groups with the low (Figure 4a) and high (Figure 4b) GO concentrations. In Figure 4a, for the GSes with low GO concentrations, the semicircular frequency behavior was clearly observed, and the diameters of the circles rapidly reduced as the GO concentration increased. This indicates that the charge conductivity (due to the resistor components in the GS) significantly increased with the higher GO concentrations. However, for the GSes with high GO concentrations, as shown in Figure 4b, the reactance changes with frequencies (toward the inductive direction) were smaller than the decreases in resistance, according to the GO concentration, meaning that the conductive current was dominant (very small resistance) in the GSes with high GO concentrations, and the phase delay of the conductive current increased in proportion to the frequency as if the inductors were connected in series.

**Figure 5:**
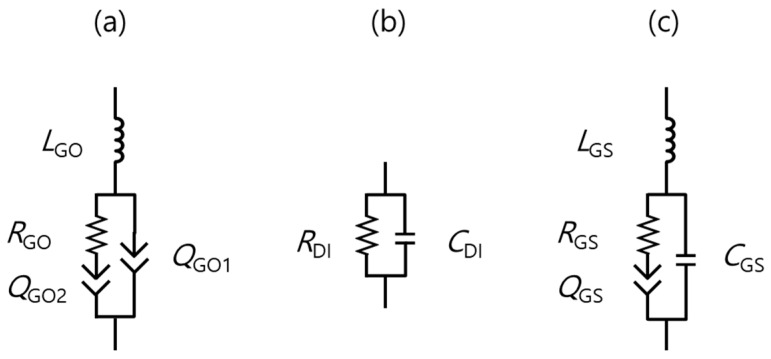
Equivalent circuit models: (**a**) stacked three-dimensional (3D) GO10; (**b**) de-ionized (DI), and (**c**) GS.

Based on the impedance analysis of Figure 2, Figure 3 and Figure 4 and our previous work [10], we proposed an equivalent GS circuit model, as shown in Figure 5. At first, the circuit model was developed for a stacked three-dimensional (3D) GO with an inductor (*L*_GO_), resistor (*R*_GO_), and two constant phase elements (CPE: *Q*_GO1_ and *Q*_GO2_), as shown in Figure 5a [10]. Generally, when circuits are not expressed as simple RC circuits, the CPE can be introduced [25] with a frequency independent *Q*-value, an imperfective resistive capacitance, and index *α* (0 < *α* < 1; *α* = 0 for a pure resistor and *α* = 1 for an ideal capacitor), as given in Equation (1).

(1) $$Z_{Q}=\;\frac{1}{Q{\left(jw\right)}^{a}}$$

In the previous work [10], the stacked 3D graphite showed an inductive conductor property, while the fully oxidized GO stack showed highly-resistive properties consisting of a large resistor and a CPE pair with a long phase delay (an inductor in series), as shown in Figure 5a. Once the oxidation was sufficiently completed, *Q*_GO1_ became similar to an ideal capacitor with an *α* of ~1. While the GOs were dispersed in DI water in this work, both of the circuit diagrams could be similar in principle. From the EIS measurements that are shown in Figure 2, the DI water was identified as the circuit of an RC pair, as shown in Figure 5b. In the circuit model of the DI water, even a very small quantity of aqua ions could contribute to the conductive current flow, and the non-conducting water served as the capacitor’s dielectric material via the water that was de-ionized for that purpose, which was shown as the EIS results of Figure 2. When considering the GO in the GS as a conductive network component and the DI water as a matrix material, we hypothesized that the equivalent circuit model of the GS could be similar to that of the GO. However, one of the GS’s CPEs (Figure 5a) was closer to an ideal capacitor because of the capacitor component in the DI water, as shown in Figure 5c.

**Figure 6:**
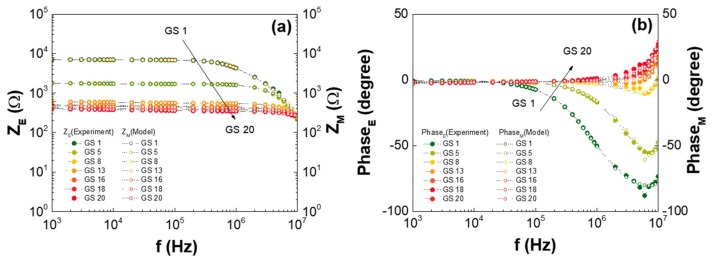
GS impedance characteristics for various GSes and GO concentrations; (**a**) magnitude and (**b**) phase of the measured and calculated impedances.

**Figure 7:**
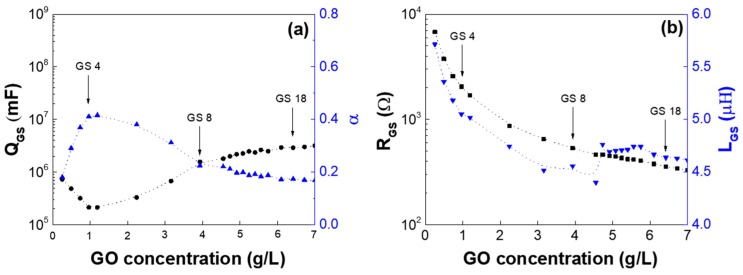
GS circuit component parameters: (**a**) constant phase elements (*Q*_GS_) with the index of *α*, and (**b**) *L*_GS_ and *R*_GS_ as a function of frequency.

Figure 6 shows the impedance characteristics of the GS samples depending on GO concentrations with frequencies, which are correlated with the modeling values. This showed that the measured magnitude (Figure 6a) and phase shift (Figure 6b) of the frequency response were well matched with the calculated responses of the circuit model of Figure 5c using the best-fitted parameters. This agreement indicates that the accuracy of the model was further verified. It could be inferred that the well-suspended GO flakes (a few hundred μm in size) in the DI water contributed to the charge conduction as the GO concentration increased in the GS regions with low GO concentrations. On the other hand, in the GS regions with high GO concentrations, as the GO concentration increased, the randomly-connected GO network and the complexity of the current-flowing mechanism retarded the phase delay of the current to the external voltage. Thus, the inductive behavior of the current could be observed at high frequencies, as shown in Figure 4b. Based on this premise, several physical parameters, including the *L*_GS_, *R*_GS_, and *Q*_GS_, with their own *α* and *C*_GS_ values, were also extracted. Their typical values are listed in Table 2 and Figure 7 in detail.

Figure 7 presents the extracted circuit parameters of the GS samples, *L*_GS_, *R*_GS_, and *Q*_GS_, and *α*, as a function of GO concentration in the GS. We note that the *C*_GS_ values are ~30 pF for all the GSes regardless of GO concentration, and we presumed that the *C*_GS_ value could be related to the volume ratio of the DI water in the GS. The results show that both *R*_GS_ and *L*_GS_ consistently decreased as the GO increased in the GS, as described for Figure 2, Figure 3 and Figure 4. Of note, the resistor, *R*_GO_, of the stacked 3D GO [10] was almost 10 times higher than that of the GS in this work. The stacked-3D GOs were closer to the capacitor than the resistor; i.e., *α*_1_ and *α*_2_ were 1.0 and 0.85, respectively. On the other hand, the GS in this work was closer to resistive (the *α* of the *Q*_GS_ was less than 0.42 in Figure 7a), even though a small amount of GO was dispersed in the DI water. We presumed that the hydroxyl/epoxide or carbonyl/carboxyl groups of the GO in the GS could be laterally connected as the GO concentration increased, and thereby contribute to the resistive path. Based on this fundamental understanding, the adsorption and desorption of molecules to the GO surfaces and their influence on the impedance of the system would be worthy of studying via a titration experiment in further work [26].

## 4. Conclusions

Oxidizing graphite and dispersing it in DI water, we formed a GS and investigated its optical and electrical properties for a range of GO concentrations (0.25 to 7.00 g/L). The transmittance of the GS become totally opaque in the visible range of 300 to 700 nm at GO concentrations exceeding 2 g/L. The electrical impedance of the GS was measured using the EIS method in the frequency range of 1 kHz to 10 MHz, and presented via both Bode and Nyquist plots. The capacitance component approached a certain constant value, but the resistance component continuously decreased as the GO concentration increased. Based on the results, we developed the equivalent GS circuit model for the various GO concentrations. The circuit model showed that the highly resistive GO became very conductive in the DI water. The findings in this work should draw new attention toward GSes and their related applications, including functional composite materials, catalysts, and filter membranes, by providing an improved electrochemical active area and improving the charge-flowing transfer properties.

## Figures and Tables

**Table 1:** Various GO concentration in GS samples.

**Low-GO Samples**	**GS1**	**GS2**	**GS3**	**GS4**	**GS5**	**GS6**	**GS7**	**GS8**	**GS9**	**GS10**	**GS11**
Concentration(g/L)	0.25	0.49	0.73	0.96	1.19	2.24	3.16	3.93	4.55	4.73	4.91
**High-GO samples**	**GS12**	**GS13**	**GS14**	**GS15**	**GS16**	**GS17**	**GS18**	**GS19**	**GS20**	**n/a**	**n/a**
Concentration (g/L)	5.08	5.24	5.40	5.56	5.75	6.10	6.42	6.72	7.00		

**Table 2:** Component values of various GSes.

Circuit Component	DI Water	GS4	GS8	GS18
*L*_GS_ (µH)	*n*/*a*	5.05	4.56	4.64
*R*_GS_ (kΩ)	102	2.05	0.53	0.36
*Q*_GS_ (mF·S^(*α*−1)^)	*n*/*a*	0.22	1.56	2.91
*α*	*n*/*a*	0.41	0.23	0.17
*C*_GS_ (pF)	28.8	30.4	28.9	30.1
